# Mutational Spectrum of Semaphorin 3A and Semaphorin 3D Genes in Spanish Hirschsprung patients

**DOI:** 10.1371/journal.pone.0054800

**Published:** 2013-01-23

**Authors:** Berta Luzón-Toro, Raquel M. Fernández, Ana Torroglosa, Juan Carlos de Agustín, Cristina Méndez-Vidal, Dolores Isabel Segura, Guillermo Antiñolo, Salud Borrego

**Affiliations:** 1 Department of Genetics, Reproduction and Fetal Medicine, Institute of Biomedicine of Seville, University Hospital Virgen del Rocío/Consejo Superior de Investigaciones Científicas/University of Seville, Seville, Spain; 2 Centre for Biomedical Network Research on Rare Diseases, Seville, Spain; 3 Department of Pediatric Surgery, University Hospital Virgen del Rocío, Seville, Spain; 4 Department of Pathology, University Hospital Virgen del Rocío, Seville, Spain; University of Hong Kong, Hong Kong

## Abstract

Hirschsprung disease (HSCR, OMIM 142623) is a developmental disorder characterized by the absence of ganglion cells along variable lengths of the distal gastrointestinal tract, which results in tonic contraction of the aganglionic colon segment and functional intestinal obstruction. The *RET* proto-oncogene is the major gene associated to HSCR with differential contributions of its rare and common, coding and noncoding mutations to the multifactorial nature of this pathology. In addition, many other genes have been described to be associated with this pathology, including the semaphorins class III genes *SEMA3A (*7p12.1) and *SEMA3D* (7q21.11) through SNP array analyses and by next-generation sequencing technologies. Semaphorins are guidance cues for developing neurons implicated in the axonal projections and in the determination of the migratory pathway for neural-crest derived neural precursors during enteric nervous system development. In addition, it has been described that increased *SEMA3A* expression may be a risk factor for HSCR through the upregulation of the gene in the aganglionic smooth muscle layer of the colon in HSCR patients. Here we present the results of a comprehensive analysis of *SEMA3A* and *SEMA3D* in a series of 200 Spanish HSCR patients by the mutational screening of its coding sequence, which has led to find a number of potentially deleterious variants. *RET* mutations have been also detected in some of those patients carrying *SEMAs* variants. We have evaluated the A131T-*SEMA3A*, S598G-*SEMA3A* and E198K-*SEMA3D* mutations using colon tissue sections of these patients by immunohistochemistry. All mutants presented increased protein expression in smooth muscle layer of ganglionic segments. Moreover, A131T-*SEMA3A* also maintained higher protein levels in the aganglionic muscle layers. These findings strongly suggest that these mutants have a pathogenic effect on the disease. Furthermore, because of their coexistence with *RET* mutations, our data substantiate the additive genetic model proposed for this rare disorder and further support the association of *SEMA*s genes with HSCR.

## Introduction

Hirschsprung disease (HSCR, OMIM 142623) is a developmental disorder occurring in 1 of 5,000 live births. HSCR most commonly presents as isolated cases and displays a complex pattern of inheritance with low, sex dependent penetrance and variable expression. It is characterized by the absence of ganglion cells along variable lengths of the distal gastrointestinal tract, which results in tonic contraction of the aganglionic colon segment and functional intestinal obstruction. Such aganglionosis is attributed to a failure of neural crest cells (NCC) to migrate, proliferate, and/or differentiate during enteric nervous system (ENS) development in the embryonic stage [1], [2].

The *RET* proto-oncogene (OMIM 164761) is the major gene associated to HSCR. *RET* has been extensively studied in HSCR patients and over 100 mutations have been identified along the gene (see Human Gene Mutation Database). However, mutations in the *RET* coding sequence account for only up to 50% or 7–20% of familial and sporadic cases, respectively [2]. The involvement of *RET* in the pathogenesis of HSCR is further supported by the existence of a specific haplotype, constituted by common *RET* polymorphisms, which seems to be responsible for the majority of sporadic forms [3], [4], [5]. This HSCR-associated *RET* haplotype is characterized by a common allele (c.73+9277T, rs2435357) within a conserved enhancer-like sequence in intron 1 (MCS+9.7) [5], [6], making a 20-fold greater contribution to risk than coding mutations [5]. It has been demonstrated a difference in ability of *SOX10* to bind to MCS+9.7 and transactivate *RET* depending on the bearing allele at such specific locus [6].

On the other hand, numerous molecular genetic studies have identified rare coding mutations in many other genes (*GDNF, NRTN, PSPN, EDNRB, EDN3, ECE1, NTF3, NTRK3, SOX10, PHOX2B, L1CAM, ZFHX1B, KIAA1279, TCF4, PROK1, PROKR1, PROKR2, GFRA1, NRG1 and SEMAs*) related to HSCR [2], [7]–[13]. However, the conventional mutations related to HSCR reported so far only explain around 5% of cases, being the vast majority of them long segment HSCR and/or total colonic aganglionosis and syndromic forms of the disease [2].

HSCR is regarded as a complex and multifactorial genetic disorder, in which the contribution of several different loci acting in an additive or multiplicative manner is usually required to cause the disease [3]. Based on this evidence, several HSCR-associated regions (9q31 [14], 19q12 [15], 3p21 [15], [16], 16q23 [17], 21q21 [18], or 4q31.3-q32.3 [19]) have been identified by genome wide linkage and genome wide association studies (GWAS), although the genes underlying such associations have not been identified yet in most of the cases.

Through a GWAS performed by the International HSCR Consortium, in which our group takes part, a significant cluster of SNPs was identified in a region on chromosome 7, containing strong association with HSCR with allelic effects independent of *RET*, that fell downstream from the protein SEMA3D (7q21.11; OMIM 609907) and upstream from SEMA3A (7p12.1; OMIM 603961) (*S. Arnold et al., 2^nd^International Symposium: Development of the ENS: Cells, signals and genes. Feb 2009*). In such study, short-HSCR trios were analyzed with the 500K SNP array platform (Affymetrix) and the *SEMA* SNPs cluster was identified and subsequently refined. The two *SEMA* family III members demonstrated very similar temporo-spatial patterns of expression throughout the colon. Notably, they were co-expressed with *RET* in this tissue, supporting the possibility that any of them might modify *RET* function in the developing ENS.

In addition, based in these findings, next-generation sequencing technologies and functional analyses have allowed the identification of semaphorins class III genes (SEMAs) *3A* and *3D* mutations potentially involved in the pathogenesis of HSCR [13].

SEMAs represent the largest family of axonal guidance cues identified so far, that provides directional information to growing axons. The role of SEMAs pattering sensory projections in the peripheral [20] and central nervous system [21] is well known. Previous studies have also suggested a role for members of the SEMA family in NCC development, proliferation, migration, and/or differentiation, which are processes related with HSCR etiology [22]–[25]. More specifically, *SEMA3A* is expressed in the mesenchyme of distal large intestine and it also acts as a repulsive signal for neurites of Remak ganglia [26]. Although ENS precursors derived from sacral NCCs express the receptor Neuropilin1 (NP1), it remains to be demonstrated if *SEMA3A* acts on migration by a direct effect on the migrating ENS precursors, or by an indirect action over extrinsic axons that accompany those cells when colonizing the bowel, and are repelled if SEMA3A is present in the outer segments of colon mesenchyme [22]. More recently, different *in vitro* and *in vivo* approaches reinforce the important role of SEMAs and their receptors as key players in the immunological and the neurological systems [27]–[29].

Furthermore, it has been proposed that increased *SEMA3A* expression may be a risk factor for HSCR pathology in a subset of HSCR patients, based on the upregulation of this gene in the aganglionic smooth muscle layer of the colon [30]. In addition, the association between two *SEMA3A* polymorphisms (rs7804122 and rs797821) and the risk of HSCR in the Northeastern Chinese population has been validated, as it was previously demonstrated in Caucasian population [31]. Based on the evidences of the implication of *SEMA* class III genes in HSCR, we have performed a screening of the coding region of *SEMA3A* and *SEMA3D* genes in a series of 200 isolated Spanish HSCR cases, to determine their mutational spectrum in our population. Three mutations, A131T-*SEMA3A,* S598G-*SEMA3A* and E198K-*SEMA3D* were further studied using an immunohistochemical approach to evaluate their expression levels in the HSCR samples. All mutants presented an increased amount of the proteins in colon tissue sections in comparison with the normal proteins, indicating the pathogenic role of these mutations.

## Materials and Methods

### Patients and Controls Subjects

In this study we have included a total of 200 Spanish HSCR patients (23% female, 77% male), and their parents when available. 180 were sporadic cases, while 20 were familial cases belonging to 13 different families. In addition, we have also analyzed a group of 200 normal controls comprising unselected, unrelated, race, age, and sex-matched individuals.

### Ethics Statement

A written informed consent was obtained from all the participants for clinical and molecular genetic studies. The study conformed to the tenets of the declaration of Helsinki as well as the requirements established by our Institutional Review Board.

### Mutational Analysis

Genomic DNA was extracted from peripheral blood leukocytes from all the individuals included in the study, using standard protocols. The mutational screening of the complete coding sequence of *SEMA3A* and *SEMA3D* was carried out by denaturing high-performance liquid chromatography in a WAVE DNA Fragment Analysis system (Transgenomic). Those fragments with aberrant profiles were subjected to sequence analysis using an ABI Prism®3730 Genetic Analyzer and the SeqScape® v2.5 software (Applied Biosystems). When a change was detected, the appropriate DNA fragment was also screened in a group of 200 normal controls to determine its allelic frequency in our population. Primers and PCR-dHPLC conditions used are avaliable under request.

### Immunohistochemistry (IHQ)

Formalin-fixed paraffin embedded (FFPE) colon tissue blocks were collected from patients with the following mutations: A131T-*SEMA3A* (both ganglionic and aganglionic sections), S598G-*SEMA3A* and E198K-*SEMA3D* (only ganglionic tissues). Unfortunately, no material from patients with R634Q-*SEMA3D* mutations was available. Four µm thick paraffin sections were dewaxed in xylene and rehydrated in a series of graded alcohols. Endogenous peroxidase activity was blocked with water containing 3% H_2_O_2_ for 30 minutes. Antigen retrieval was done by microwaving using citrate phosphate buffer (pH 6.0). Sections were incubated at 4°C overnight with the primary antibodies anti-*SEMA3A* (1∶50 dilution, anti-rabbit, AntibodyBcn, Barcelona, Spain) and anti-*SEMA3D* (1∶5 dilution, anti-rabbit, Novus Biologicals, Cambridge, UK). After several washes in Tris buffer, peroxidase-labelled secondary antibodies and 3,3′-diaminobenzidine were applied to develop immunoreactivity, according to manufacturer’s protocol (EnVision; Dako, Glostrup, Denmark). The slides were then counterstained with hematoxylin and mounted in DPX (BDH Laboratories, Poole, UK). Colon tissue section in which primary antibody was omitted was used as negative control.

## Results and Discussion

Members of SEMA class III protein subfamily are inhibitory axon guidance molecules, which could help to determine the axonal projection patterns of several neurons and ganglia throughout the central and peripheral nervous system [32]. Moreover, *SEMA3A* has been related to HSCR through different approaches [13], [30], [31].

We have detected a total of 56 sequence variants in the mutational screening of *SEMA3A* and *SEMA3D* genes (Tables 1 and 2). The most interesting finding among these results was the detection of four missense variants (*SEMA3A*: A131T, S598G; *SEMA3D*: E198K, R634Q) in heterozygosis. Two of them were already described (A131T-3A with a MAF = 0,01 and R634Q-3D with a MAF = 0,0093) in public databases (1000 Genomes, EVS and NCBI) but the other two (S598G-3A and E198K-3D) were novel. All four variants are located in the coding region of both SEMA proteins and found exclusively in HSCR series but absent in the control population (Table 3). They were detected with a mutational frequency range of 0.005%-0.01%, which was comparable with the percentages of *SEMA* mutations previously described in Caucasian population (0.004%–0.011%) [13].

**Table 1:** *SEMA3A* sequence variants detected in the current study.

Nucleotide Change	Aminoacid Change	Novel/Described	Allelic frequency (%) in control population
c.112+52del		Novel	0
c.112+52 C>G		Novel	0
c.112+63 T>C		rs13231702	0
c.113-110 A>C		rs12671857	52.2
c.113-42 G>C		rs17241389	0
c.201T>C	S67S	Novel	1
c.267A>G	Q89Q	Arnold et al., 2009	0
c.270+96A>G		Novel	0
c.333+84T>A		Novel	0
c.333+92G>T		rs6955597	0.3
c.334-24delTT		Novel	0
**c.391G>A**	**A131T**	**rs143007146**	
c.453+24A>G		rs1990044	47.9
c.547+167G>A		rs2527039	15.8
c.548-45C>T		Novel	0
c.548-67T>C		Novel	1
c.548-79G>C		Novel	0
c.548-92 T>C		Novel	0
c.668-199_201del		Novel	2.5
c.668-138A>T		Novel	0
c.668-20 C>T		rs2272221	10.5
c.668-14 T>A		rs2272222	2.2
c.705T>C	S235S	rs34541339	6.8
c.732C>T	Y244Y	Novel	0
c.811-139C>T		Novel	0
c.945C>T	N315N	Novel	0
c.1140+46C>G		Novel	0
c.1302T>C	I434I	Novel	15
c.1303G>A	V435I	Arnold et al., 2009	3
c.1361-52T>A		rs10250165	23.8
c.1361-14A>G		rs3735513	14.7
c.1453-9delA		Novel	0
c.1495+132A>C		rs17246251	61.5
c.1563G>C	G521G	rs10487865	0
c.1652-85insTA		Novel	0
c.1653-6C>T		rs701320	84
**c.1792A>G**	**S598G**	**Novel**	**0**
c.1860+30A>G		rs7809708	26.5
c.2151A>G	T717T	rs797821	32.5

Compilation of all the *SEMA3A* sequences variants found by dHPLC. Note that the missense variants are shaded.

**Table 2:** *SEMA3D* sequence variants detected in the current study.

Nucleotide Change	Aminoacid Change	Novel/Described	Allelic frequency (%) in control population
c-151-158_154del		Novel	1
c.376-32G>A		Novel	0
c.496-12T>C		Novel	0
c.589+37G>A		rs17159594	0
c.590-33T>A		Novel	3.5
c.718+26T>C		Novel	0
c.636C>T	D212D	Novel	0
**c.592G>A**	**E198K**	**Novel**	**0**
c.861+67_71del		rs56131427	4.8
c.1545+91G>A		rs7780132	28.3
c.1546-9 G>A		Novel	0
c.1578C>T	L526L	rs17559084	43.8
c.1703+28G>C		rs6468008	27
c.1843C>A	P615T	Novel	0.5
**c.1901G>A**	**R634Q**	**TMP_ESP_7∶84636125** *	**0**
c.1906+147T>C		Novel	0
c.2103G>T	K701Q	rs7800072	35.8

Compilation of all the *SEMA3D* sequences variants found by dHPLC. Note that the missense variants are shaded.

* Source: Ensemble.

**Table 3:** *SEMA3A* and *SEMA3D* missense variants detected in isolated HSCR patients.

Mutation information				Patients information			
Gene	Nucleotide Change	Aminoacid Change	Protein domain	Gender	Length of aganglionosis	Inheritance	Other mutational events in the patient
SEMA3A	c.391G>A	A131T	SEMA	Male	Not available	Paternal	*RET* enhancer mutation in homozygosis
SEMA3A	c.391G>A	A131T	SEMA	Male	Not available	Maternal	*RET* R313W mutation inherited from the father
SEMA3A	c.1792A>G	S598G	Ig-like	Female	Sigmoid	Data not available* ^1^	*RET* W543R mutation* ^1^
SEMA3D	c.592G>A	E198K	SEMA	Male	Rectosigmoid	*De novo* or paternal* ^2^	*RET* enhancer mutation inherited from the mother
SEMA3D	c.1901G>A	R634Q	Ig-like	Male	Hepatic Flexure	Maternal	*EDNRB* K15X mutation inherited from the father
SEMA3D	c.1901G>A	R634Q	Ig-like	Male	Sigmoid	Maternal	*RET* enhancer mutation in homozygosis

Detailed information regarding the four *SEMA3A* and *SEMA3D* missense variants detected in isolated HSCR patients.

* ^1^Both *RET* and *SEMA3A* mutations could not be verified to be inherited or *de novo* events, since DNA samples from the parents of this patient were not available.

* ^2^The E198K-*SEMA3D* mutation could not be verified to be paternally inherited or a *de novo* event, since paternal DNA was not available.

**Figure 1:**
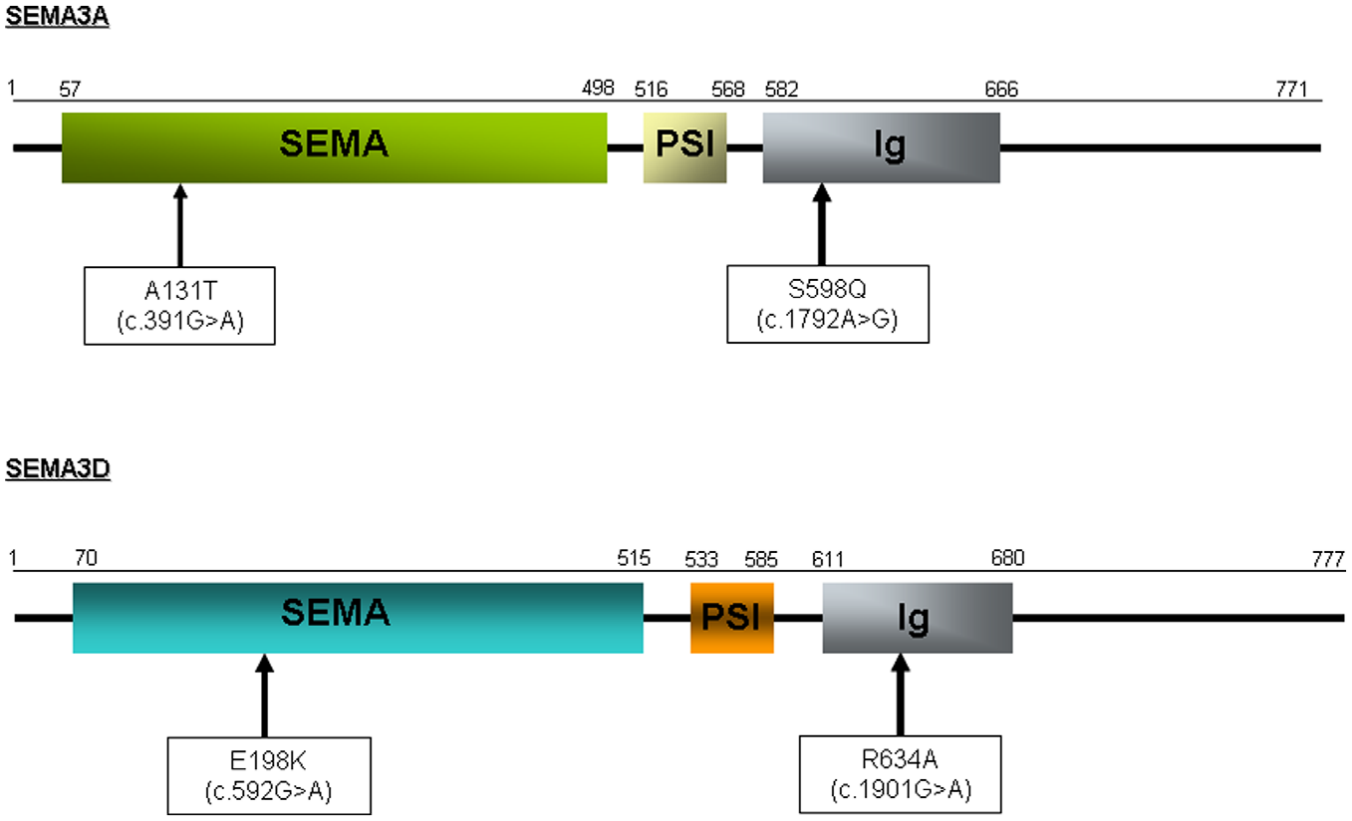
Schematic domain location of the mutations analyzed in human Sema3A and Sema3D proteins. An N-terminal seven-bladed β-propeller Sema domain followed by a cysteine-rich PSI (plexin, semaphorin, integrin) domain is a signature feature in the ectodomains of semaphorin and plexin family members. The composition of the remainder of the semaphorin ectodomain varies according to class: the ectodomains of the secreted class 3 semaphorins contain an Ig (immunoglobulin-like) domain and a basic C-terminal tail.

**Figure 2:**
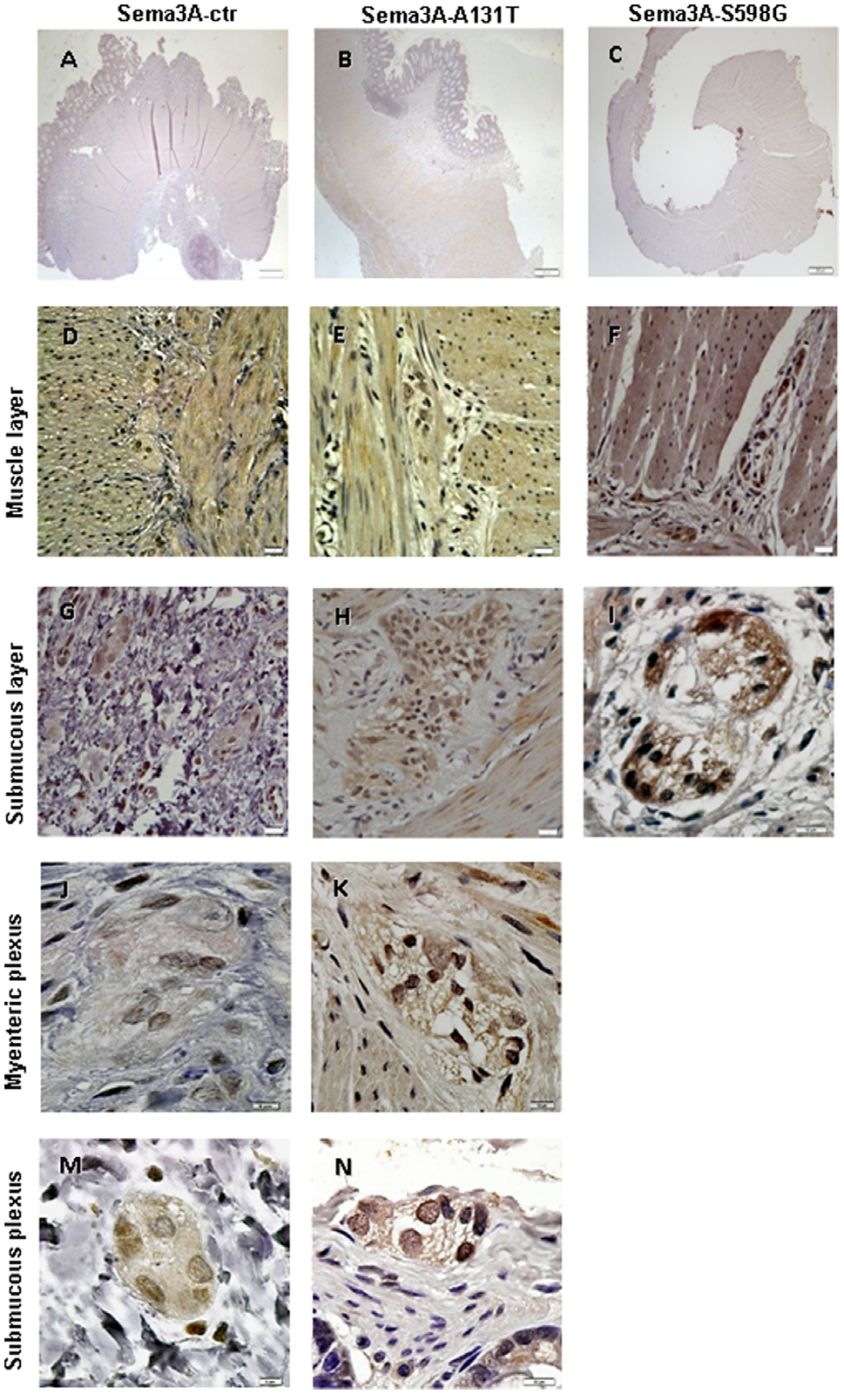
Immunostaining of Semaphorin 3A in colon from controls and HSCR patients. The SEMA3A staining illustrated that the expression was present at smooth muscle (D, E, F) and submucous (G, H, I) layers, as well as in myenteric (J, K) and submucous plexuses (M, N) either in normal colon (A, D, G, J, M) and patients with A131T-3A (B, E, H, K, N) and S598G-3A mutations (C, F, I). The FFPE tissue block from patient with S598G had no all tissue layers. Scale bars: A–C = 200 µm and the rest of pictures = 10 µm.

**Figure 3:**
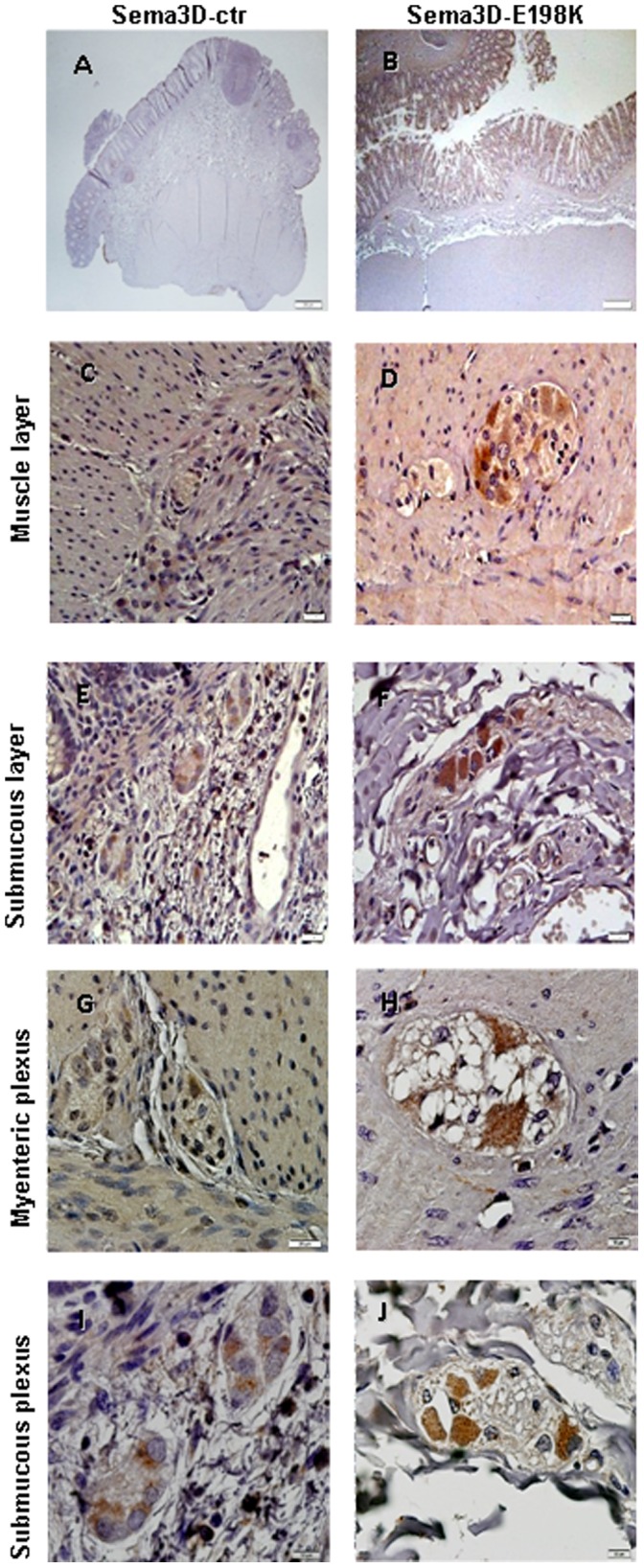
Immunostaining of Semaphorin 3D in colon from controls and HSCR patients. The SEMA3D staining illustrated was present at smooth muscle (C, D) and submucous (E, F) layers, as well as in myenteric (G, H) and submucous plexuses (I, J) either in normal colon (A, C, E, G, I) and patients with E198K-3D mutation (B,D, F, H, J). Scale bars: A, B = 200 µm and the rest of pictures = 10 µm.

There are several evidences that led us to propose the four missense variants in two *SEMAs* genes (*SEMA3A*: A131T, S598G; *SEMA3D*: E198K, R634Q) as mutations associated to HSCR. First, the mutations were detected in two main protein domains (Figure 1): the SEMA domain, which is important for protein-protein interaction (A131T*-SEMA3A* and E198K-*SEMA3D*) and the domain implicated in receptor binding (S598G-*SEMA3A* and R634Q-*SEMA3D*). Second, *in silico* predictions (SIFT, Polyphen) had shown the damaging effect of those four mutant proteins. Here, we have also partially characterized the mutations A131T-*SEMA3A* and S598G*-SEMA3A* and E198K-*SEMA3D* by immunohistochemistry. In histological analyses, both *SEMA3A* (Figure 2) and *SEMA3D* (Figure 3) were expressed in the ganglion cells of the myenteric and submucosal plexuses as well as in the smooth muscle layers of the ganglionic colon of our patients and in the control samples.

**Figure 4:**
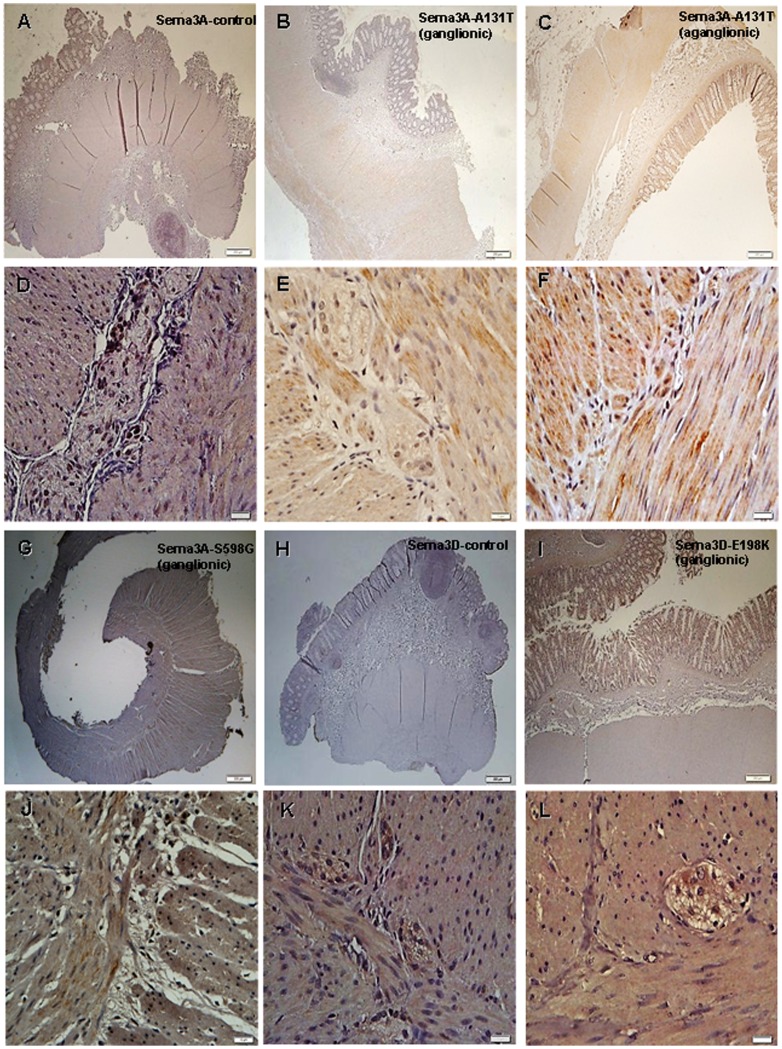
Immunohistochemical detection of A131T-*SEMA3A,* S598G-*SEMA3A* and E198K-*SEMA3*D mutant proteins in FFPE colon samples. Immunohistochemistry analysis of A131T-3A, S598G-3A and E198K-3D mutant proteins in smooth muscle layer of control samples (3A: A, D; 3D: H, K) and colon tissue from patients with A131T-3A (ganglionic: B, E; aganglionic: C, F), S598G-3A (G, J) and E198K-3D mutations (I, L) was performed. Scale bars: A, B, C, G, H, I = 200 µm and the rest of the pictures = 10 µm.

Regarding the A131T-*SEMA3A* mutation, we obtained both ganglionic and aganglionic segments from the patient. A highly expressed mutant SEMA3A protein in the smooth muscle layers in the aganglionic segment was detected. This increased amount of protein seemed to be higher than the increase found in both the corresponding ganglionic segment and the control sample (Figure 4). This is in accordance with previously published results for the wild type SEMA3A protein, that it is upregulated in the aganglionic colon tissue [30].

In the case of S598G-*SEMA3A* and E198K-*SEMA3D* mutants, a more intense immunostaining in all ganglionic tissue layers in comparison with the control sample was detected (Figure 4).

Based on the increased amount detected of both SEMA3A and SEMA3D proteins in colon tissue of HSCR patients, we propose that those variants results in the accumulation of SEMAs proteins leading to impaired enteric axonal projection during NCCs migration and/or differentiation, with functional consequences in ENS formation. Given the role of SEMAs as crucial modulators during embryonic development [32], abnormal SEMAs expression or function may have a pleiotropic effect, as it has been observed in mouse null mutants of genes encoding *SEMA3A* and its receptor *NP1* [33]. This hypothesis fits with the lack of null mutations in our mutational screening, being more plausible that the missense mutations detected on *SEMA3A* and *SEMA3D* genes cause a more subtle effect on protein function than null mutations do. Thus, we propose that the genetic background of the individual in combination with the presence of those hypomorphic mutations in the *SEMA* genes would generate the specific phenotype observed in our patients, in accordance with the additive model previously proposed for HSCR [34]. We failed to detect any other coding mutation in the previously associated HSCR genes in the patients with *SEMA* mutations (data not shown), except for *RET* and *EDNRB* genes (Table 3), although we cannot discard the contribution of additional mutational events in still unidentified HSCR genes.

It is worth of mention the high incidence of co-occurrence of coding *RET* mutations and *SEMA3A* mutations in our series of patients, as 50% of the patients with mutations on this *locus* present coding mutations in *RET*. All patients carrying mutations either in *SEMA3A* or *SEMA3D* genes have inherited either *RET* enhancer variant or a coding mutation associated to HSCR.

It is well established that activation of *RET* is essential for proliferation, migration and differentiation of enteric neural precursors [35]. The vast majority of enteric neurons and glial cells arise from vagal NCCs, although sacral NCCs also give rise to some neurons and glia, principally in the distal hindgut, after the caecum [36], [37]. Migration of enteric precursors derived from vagal NCCs, within the colon has been shown to depend mainly on the proliferation at the migration wave front, rather than on the presence of factors that promote migration [38]. For that reason, mutations in *RET* cause a failure of those cells to colonise the distal colon in an appropriate number to form the enteric ganglia, leading to HSCR [2]. The expression of *GDNF* in the hindgut is lower than in the caecum [39] and therefore many authors have proposed that migration and proliferation of sacral NCCs is less dependent on GDNF. In addition, sacral NCCs are able to migrate through pre-caecum colon in a slower manner compared to vagal derived cells [22], [39]–[42]. In such scenario, we propose that the absence of enteric ganglia in the distal part of the bowel, due to a fail of vagal NCCs, could be rescued by the migration and differentiation of sacral NCCs. It has been demonstrated that *SEMA3A* regulates the entry of extrinsic axons into the hindgut and also sacral NCCs, as they accompany those axons when migrating to the colon. In fact, defects on *SEMA3A* expression impair the entry of sacral enteric precursors in the hindgut [22]. For that reason, we suggest that a fail on sacral NCCs to colonize the hindgut in the appropriate moment, due to the presence of mutations in *SEMA3A*, would results in a more dramatic phenotype in patients also harbouring mutations in *RET*, as *SEMA3A* has no effects on vagal NCCs [22]. In agreement with this hypothesis, we did not observe an enteric phenotype in family members of our HSCR patients with isolated mutations in *RET* or *SEMA3A*. Segregation analysis for these mutations in both genes together with the available data of mutations in other genes associated to HSCR, led us to speculate with the potential synergistic effect of mutations in both *RET* and *SEMA* genes. We believe that the major contributing mutation could be either on *RET* or *SEMA*, or in both genes at the same time alone or together with some other mutations in unknown genes, due to the complexity of the genetic basis of HSCR and the additive model proposed for the disease. Future analyses will be needed to clarify the role of both genes.

In summary, in this study we have analyzed three aminoacidic substitutions, A131T-*SEMA3A,* S598G-*SEMA3A* and E198K-*SEMA3D*. These variants result in an increase of SEMA proteins levels in the HSCR colon tissue. Our findings further support the functional implication of SEMAs as signalling molecules in the development of ENS and indicate a synergistic effect of mutations in both *SEMA and RET* genes, to influence the phenotype of our HSCR patients.
